# Adapting Drug Approval Pathways for Bacteriophage-Based Therapeutics

**DOI:** 10.3389/fmicb.2016.01209

**Published:** 2016-08-03

**Authors:** Callum J. Cooper, Mohammadali Khan Mirzaei, Anders S. Nilsson

**Affiliations:** Department of Molecular Biosciences, The Wenner-Gren Institute, Stockholm UniversityStockholm, Sweden

**Keywords:** bacteriophage, phage therapy, adaptive pathways, alternative licensing, pharmaceutical regulation

## Abstract

The global rise of multi-drug resistant bacteria has resulted in the notion that an “antibiotic apocalypse” is fast approaching. This has led to a number of well publicized calls for global funding initiatives to develop new antibacterial agents. The long clinical history of phage therapy in Eastern Europe, combined with more recent *in vitro* and *in vivo* success, demonstrates the potential for whole phage or phage based antibacterial agents. To date, no whole phage or phage derived products are approved for human therapeutic use in the EU or USA. There are at least three reasons for this: (i) phages possess different biological, physical, and pharmacological properties compared to conventional antibiotics. Phages need to replicate in order to achieve a viable antibacterial effect, resulting in complex pharmacodynamics/pharmacokinetics. (ii) The specificity of individual phages requires multiple phages to treat single species infections, often as part of complex cocktails. (iii) The current approval process for antibacterial agents has evolved with the development of chemically based drugs at its core, and is not suitable for phages. Due to similarities with conventional antibiotics, phage derived products such as endolysins are suitable for approval under current processes as biological therapeutic proteins. These criteria render the approval of phages for clinical use theoretically possible but not economically viable. In this review, pitfalls of the current approval process will be discussed for whole phage and phage derived products, in addition to the utilization of alternative approval pathways including adaptive licensing and “Right to try” legislation.

## Introduction

**Figure 1:**
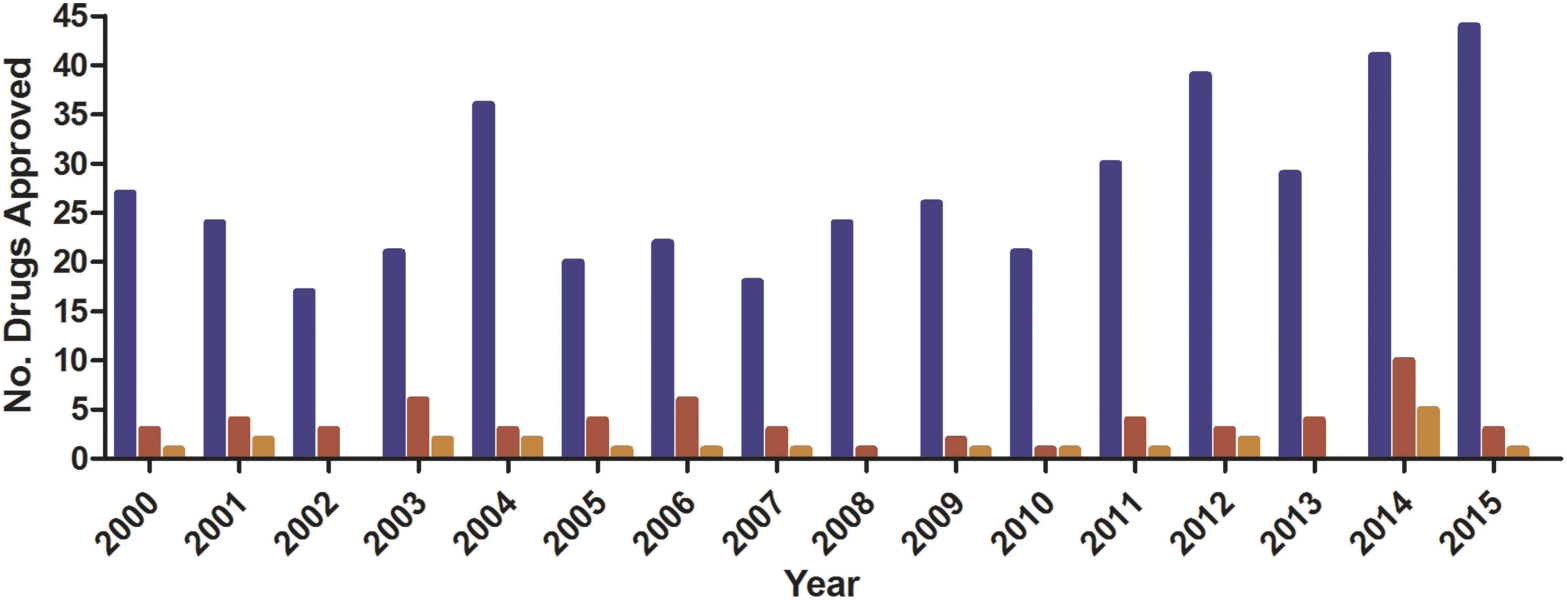
**FDA Novel Drug Approval 2011–2015**. Data obtained from (http://www.fda.gov/Drugs/DevelopmentApprovalProcess/DrugInnovation/ucm430302.htm). 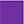, Total; 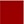, anti-infective; 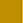, anti-bacterial.

The discovery of penicillin in 1928 heralded a dynamic shift in modern medicine with antibiotics quickly becoming one of the linchpins of modern medicine (Zaffiri et al., 2012). However, in the 1960s, the “golden era” of the identification of novel antibiotics ended with modern development focusing on the modification of existing drugs (Nathan and Cars, 2014) with only four multinational pharma companies maintaining antibiotic divisions (Fair and Tor, 2014). This lack of interest is not only due to the difficulties in discovering new antibiotic classes but also decreasing financial returns within drug development (Scannell et al., 2012). It is particularly true for anti-infective agents, where a median 10 day drug-treatment costs ~US$ 85 for non-HIV anti-microbial drugs compared to ~US$ 848 for anti-neoplastic drugs (Falagas et al., 2006). When compared to the total number of drugs granted regulatory approval, anti-infective drugs represent due to a poor return on overall investment which stifles their development (Figure 1; Piddock, 2012).

Despite public concern about increasing levels of antibiotic resistance, antibiotic consumption continues to increase, particularly in the BRIC (Brazil, Russia, India, China) nations (Van Boeckel et al., 2014). The ability to obtain antibiotics without prescription, their subsequent misuse by patients (Li, 2014), and the continued use of antibiotics as a growth promoter in agriculture (Cully, 2014), has contributed to an increase in the number and scale of multi-drug resistant infections (Molton et al., 2013). Increased consumption and fervid media reporting has generated huge interest in the development of new antibacterial agents and has led to the formation of a number of working groups, such as the US Food and Drug Administration's (FDA) Antibacterial Drug Development Task Force (http://www.fda.gov/Drugs/DevelopmentApprovalProcess/DevelopmentResources/ucm317207.htm; accessed 13th June 2016) or the Biotechs from Europe innovating in Anti-Microbial resistance (BEAM) Alliance (http://beam-alliance.eu/; accessed 13th June 2016). These groups have called for financial incentives and patent extensions to be applied to antibacterial drug development in order to stimulate research (Sonderholm, 2009; Laxminarayan and Powers, 2011; Wise, 2015).

Interest in phage therapy (i.e., the clinical use of bacteriophage based therapeutic products in humans), has been traditionally confined to academic groups and a few clinical centers in Eastern Europe, most notably the ELIAVA Institute in Tbilisi, Republic of Georgia. However, the specificity of phages and the enormous variation in human—bacteria—phage combinations will lead to an immense number of obligatory clinical trials if they are to be considered as a viable alternative to antibiotics. This constitutes one of the primary obstacles for the industrial development of phage based therapeutic products, in addition to concerns over intellectual property protection. Nevertheless, commercial interest has been piqued in the form of small BioPharma companies (Table 1), but significant interest from multinational pharmaceuticals is still lacking. These small BioPharma companies have enabled a number of commercial phage products to be approved for use in reducing food contaminants (Endersen et al., 2014), but widespread use of phage therapeutics in humans remains elusive in the West (Kingwell, 2015).

**Table 1:** **Summary of current phage based products in development for the treatment of human disease**.

**Company**	**Product**	**Type**	**Target**	**Application**	**Company website**
Micreos	Staphefekt	Endolysin	*S. aureus*	Topical	https://www.staphefekt.com/en/newspublications
Intralytix	ShigActive	Phage	*Shigella*	Ingested	http://www.intralytix.com/index.php?page=prod
AmpliPhi	AmpliPhage-001	Phage	*Ps. aeruginosa*	–^a^	http://www.ampliphibio.com/product-pipeline.html
	AmpliPhage-002		*S. aureus*	Topical	
	AmpliPhage-004		*C. difficile*	–	
Technophage	TP-102	Phage	–^a^	Ulcers	http://www.technophage.pt/index.php/r-d/product-pipeline
	TP-122		–^a^	Respiratory	
	TP-132		–^a^	–^a^	
	TP-107		–^a^	Topical	
Pherecydes Pharma	PP021	Phage	*E. coli*	Burn and Skin	http://www.pherecydes-pharma.com/pipeline.html
	PP1131 PP1231		*Pseudomonas*	Burn, Skin, and Respiratory tract infection	
	PP2351		*Staphylococcus*	Bone, Joint, and Prosthesis	
Avid Biotics	Pyocin	Phage Derived	*E. coli*	Diarrheal and food poisoning	http://www.avidbiotics.com/programs/
	Avidocin		*C. difficile*	–^a^	
	Pyocin		*Pseudomonas*	–^a^	
	Purocin		*Salmonella*	Food poisoning	
	Purocin		*Listeria*	Food poisoning	
ContraFect	CF-301	Phage Derived Lysins	*S. aureus*	–^a^	http://www.contrafect.com/pipeline/overview
	CF-303		*S. pneumoniae*	–^a^	
	CF-304		*S. faecalis* and *E. faecium*	–^a^	
	CF-305		*S. agalactiae*	–^a^	
	CF-306		*B. anthracis*	–^a^	
	CF-307		Group B Streptococcus	–^a^	

a Information not available.

The societal need for new antibacterial agents, and the knowledge that phage therapy may work in practice, requires the engagement of commercial entities to further develop phage based products rather than proceeding as a purely academic enterprise. However, what appears to limit the development of phage products for human use is primarily associated with development costs and regulations. Through the application of new or refined regulations the development of phage based pharmaceutical products may become faster and more commercially attractive for companies.

In this article, the current requirements for the development and approval of new antibacterial drugs are described with emphasis placed on the challenges faced by phages and phage based products. Potential alternative or additional approval pathways within existing and proposed legislation and how phage therapy could benefit from these pathways are also discussed.

## The revival of phage therapy

Since the early part of the 20th Century, bacteriophages have been used to treat a range of different bacterial infections (Kutter et al., 2010). However, since the introduction and success of antibiotics in the mid-20th century, interest in phages as antimicrobial agents within Western Europe and the US has waned. Increasing problems with antibiotic resistant bacterial infections has led to alternative strategies being sought. This has in turn revitalized research into bacteriophages and their derived products as antibacterial agents (Oliveira et al., 2015). Phage therapy possesses advantages and disadvantages when compared to conventional antibiotics. These advantages include the ability of phages to self-replicate in the presence of a suitable bacterial host. They act with minimal disruption to the local microbiota and are relatively easy to isolate from environmental sources, while the limited host range of lytic phages may detract from their overall clinical usefulness. Although the advantages and disadvantages of phage therapy have been briefly highlighted here, they are discussed extensively elsewhere (Loc-Carrillo and Abedon, 2011; Nilsson, 2014; Kutter et al., 2015).

Virulent phages have been isolated from a variety of environments and proven *in vitro* to be efficient against a large number of bacterial species (Mattila et al., 2015; Salem et al., 2015; Sauder et al., 2016). *In vivo* testing has shown that phages can be used to treat various types of infections in animal models (Hawkins et al., 2010; Dufour et al., 2015; Ghorbani-Nezami et al., 2015; Holguín et al., 2015; Galtier et al., 2016) and also in humans (Kutter et al., 2010; Abedon et al., 2011; Chanishvili, 2012; Rose et al., 2014; Abedon, 2015). Results from these more recent *in vitro* and in *vivo* trials have led to a deeper understanding of the unique nature of phage therapy but have also highlighted the need for further research into their pharmacokinetics and pharmacodynamics (PD/PK).

Often compared to conventional antibiotics in the lay press, the capability to kill bacteria is the only similarity that whole phages and antibiotics share. Therefore, whole phage therapy is often complicated by additional factors and as such possesses unique pharmacokinetics and pharmacodynamics that remain poorly understood. Amongst these unique characteristics is the poor diffusion of phages as the result of their immense size when compared to antibiotics (~10^6^ times larger). This means that whole phages cannot be administered in high concentrations (>10^10^ PFU). In order to provide an equivalent amount of “drug” compared to a 10 day course of penicillin (assuming equal minimum inhibitory concentrations and non-replicating phages) over 100 Kg of phages would be required (Bancroft and Freifelder, 1970; Nilsson, 2014). This lack of diffusion and restricted dosage concentration can be offset by the ability of phages to replicate upon finding their target organism.

As with all antimicrobial agents, the ever present shadow of resistance is particularly relevant to whole phage therapies where bacterial exposure to phages provides a co-selective pressure to develop and evade resistance. In the case of conventional antibiotics, targets are often essential metabolic functions, while phages and phage derived products (e.g., endolysins) primarily target surface structures, among the most rapidly changing features in bacteria. The epoch spanning co-evolutionary arms race between phages and bacteria have also resulted in the development of a number of distinct and constantly evolving anti-phage systems, most famously CRISPR-Cas systems (Horvath and Barrangou, 2010), to protect bacteria from infection by phages. In addition to CRISPR-Cas systems, other bacterial resistance mechanisms exist including phage exclusion and restriction modification systems, and have been discussed extensively elsewhere (Hyman and Abedon, 2010). While such systems present a threat to the overall efficacy of a whole phage therapeutic, they are not universally distributed in bacterial species (Grissa et al., 2007; Burstein et al., 2016) and phages also develop counter measures to these resistance mechanisms (Maxwell, 2016).

Although humans are routinely exposed to phages on a daily basis, concern persists over their immunogenicity and overall safety, presenting an additional stumbling block for the adoption of phage therapy. High doses of phage proteins can elicit unwanted side effects from stimulation of the immune system (Gorski et al., 2012; Dabrowska et al., 2014). Due to their classification as biological therapeutics (Rose et al., 2014), both whole phage therapies and therapies based on phage derived products will need to be manufactured under current good manufacturing practices (cGMP) and also adhere to current pharmacopeia requirements that are based on the type of application. This will require not only large scale manufacturing in inert suspension media, something being addressed by small biopharma, but also production of ultrapure preparations conforming to strict endotoxin requirements (<0.5 EU/mL for subcutaneous injections).

## Current clinical trials regulation

The regulatory foundation for clinical studies and clinical trials in humans is to ethically establish the potential toxicity, efficacy and side effects of new drugs and to prioritize the health of the participants over the generation of results. It is equally important that sufficient data support the claim of potential benefits and that these benefits outweigh anticipated risks. Clinical studies and trials should be carried out in a scientifically correct and transparent manner, be designed to result in trustworthy data and assess the pharmacological properties of the new drug in a stepwise process adapted to available information.

## Pre-clinical testing

In the current paper, a number of points will be discussed that specifically impact upon individual licensing pathways. In addition, there are a number of pre-clinical testing issues which need to be addressed prior to use in patients regardless of the approval process. These include the need to develop standard testing protocols such as those found in antibiotic (e.g., BSAC or EUCAST; Brown et al., 2016) or microbicide biocide testing (e.g., ASTM E2197) to ensure consistency in results. There is an ongoing shift from classical qualitative assays such as the spot test (host range is assessed by plaque formation) to more quantitative methods such as the efficacy of plating (number of plaques on a target strain compared to number of plaques on the routine host; Khan Mirzaei and Nilsson, 2015), protocols still vary between laboratories. The creation of international standards would ensure the reliability and reproducibility of data.

Standard “efficacy” criteria are utilized by companies seeking to claim activity for chemical microbicides against a particular pathogen (e.g., a defined strain of *S. aureus* as an analog for MRSA). These activities are often under defined environmental and test conditions utilizing a reference strain as the target (e.g., ASTM E2197)^1^. Currently no such standardized criteria exist for whole phages. Although whole phages are unlikely to achieve such large reductions in short time periods (usually ≥5log_10_ reduction in <5 min), suitable criteria could be established. These criteria could be based upon a defined lower kill level, the persistence of antibacterial activity over prolonged periods of time, or other virulence characteristics (Borysowski et al., 2014). Such criteria would enable multiple libraries based on lytic activity to be assembled for custom made therapies.

## Classical clinical trials

Clinical trials in the United States must be carried out in accordance with laws in the United States Code, title 21, chapter 9; the Federal Food, Drug and Cosmetic Act and in particular part A of subchapter V: Drugs and Devices (http://www.fda.gov/RegulatoryInformation/Legislation/FederalFoodDrugandCosmeticActFDCAct/FDCActChapterVDrugsandDevices/default.htm#Part_A; accessed 13th June 2016) under the jurisdiction of the FDA and have influenced the regulations of many other countries due to their comprehensive nature. Within the EU, clinical trials are currently performed in accordance to the Clinical Trials Directive (EU-CTD). This should be superseded in 2016 by the simplified and updated Clinical Trials Regulation (EU-CTR; http://ec.europa.eu/health/human-use/clinical-trials/directive/index_en.htm; accessed 13th June 2016), allowing a single application in one member state to apply to all EU member states which would participate in the trial.

**Figure 2:**
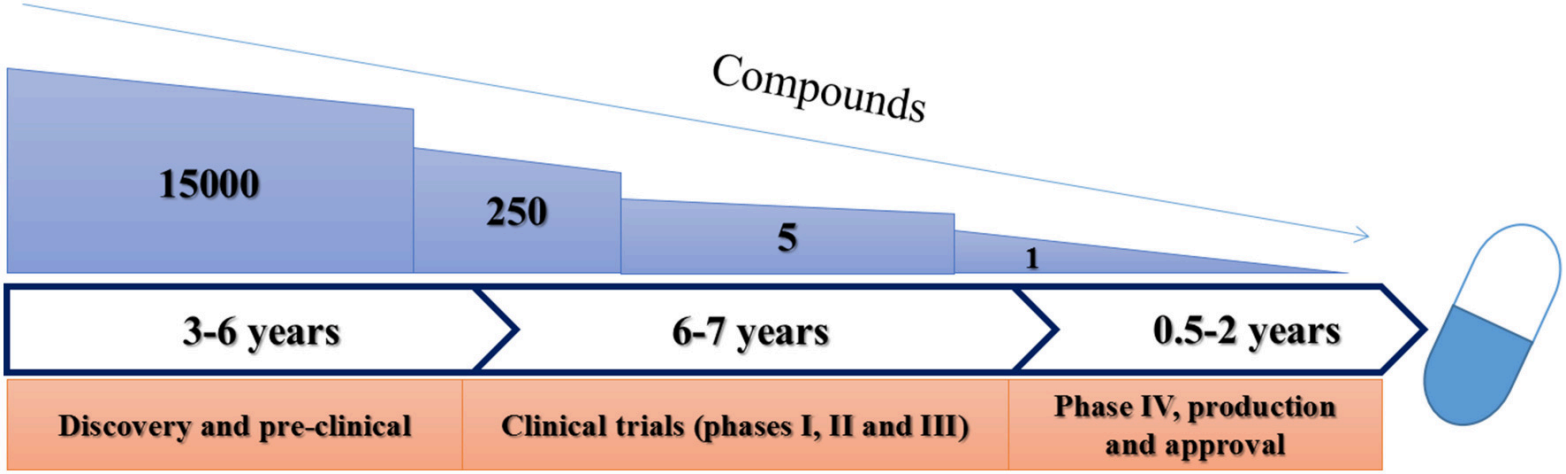
**Schematic representation of current FDA approval procedures for anti-infective drugs**.

Clinical trials are often broken into four distinct phases (Figure 2) following successful pre-clinical studies. These phases increase in complexity and size as a product moves closer to approval. Should approval be granted, all products are then subjected to rigorous routine review as they are used. These stages have been reviewed in detail elsewhere (Pocock, 1983) and are summarized in Table 2.

**Table 2:** **Summary of clinical trials testing requirements**.

**Phase**	**Aim**	**Cohort size**	**Notes**
Phase I	Determination of safety	20–100	• 1st in human studies using healthy volunteers • Evaluation of dosing while monitored • Determination of adverse effects
Phase II	Determination of efficacy	100–500	• Determination of efficacy in target population • Evaluation of side effects
Phase III	Confirmation of efficacy	1000–5000	• Verification of efficacy in target population • Evaluate rarer side effects • Comparison to gold standard treatment
Phase IV	Safety surveillance	–^a^	• Monitoring of routine use to ensure no adverse side effects

Adapted from (Pocock, 1983).

a Not applicable.

It is estimated that ~US$2.6 billion is required to successfully go from concept to an approved drug (Mullard, 2014). Although some of the initial screening has been taken up by academia (mainly pre-clinical and development work), a significant investment of time and resources is still required from pharmaceutical companies. When compared to drugs developed to treat chronic conditions (e.g., statins), the development of antibacterial agents is economically unviable, due to their comparatively short usage time.

In response to the pressing need to develop new, safe and effective antibacterial agents, additional legislation has been introduced which attempts to modify the approval process, including limited population approval and the incentivization of antibacterial drug development (Brown, 2013; Bax and Green, 2015).

## The application of whole phage and phage derived products to “classical” clinical trial scenarios

Both whole phages and their derived products will be subjected to the same rigorous clinical trials process as antibiotics. They are also classified as “Therapeutic biological products” and thus subject to the Food, Drug, and Cosmetic Act (http://www.fda.gov/RegulatoryInformation/Legislation/FederalFoodDrugandCosmeticActFDCAct/FDCActChapterVDrugsandDevices/default.htm#Part_A; accessed 13th June 2016) and also the Public Health Service Act (http://www.fda.gov/RegulatoryInformation/Legislation/ucm148717.htm; accessed 13th June 2016) and under EU Directive 2001/83/EC (Rose et al., 2014; Pelfrene et al., 2016) and would require additional controls over the manufacturing process. Should any changes be made to the manufacturing process, extensive comparability testing would be required to confirm the consistency of the product (Chirino and Mire-Sluis, 2004). Regulators, such as the EMA, are aware of the additional issues faced by phage therapeutics and believe that dialogue with developers will contribute toward a solution (Kingwell, 2015).

For ease of reference, a number of assumptions have been made in the current article as follows:

- A single strictly virulent phage or phage derived product (e.g., endolysin) is selected from pre-clinical studies which produces a suitable level of bacterial reduction for the intended application.
- The phage or phage derived product has been subjected to appropriate pre-clinical *in vivo* testing to determine the toxicity, immunogenicity, and dosing of the treatment.
- All products should be produced according to cGMP, as determined by local regulators, as well as being soluble in, and compatible with, commonly used physiological solutions (e.g., saline) or other physiologically inactive media.
- All components of a phage or phage product cocktail must have been shown to be acceptable as stated above for single phages. In addition, the dynamics between the individual components of the cocktail should also be assessed prior to use.

Initial clinical testing (Phase I) will not vary greatly between antibiotics, whole phages or their derived products and as such should be relatively simple to perform with the appropriate approvals. The routine exposure of humans to phages provides the immune system with a low level of circulating phage-specific antibodies (Kucharewica-Krukowska and Slopek, 1987) and subsequent exposure as part of a therapy may compound this. Indeed a number of *in vitro* and *in vivo* studies have shown that phages stimulate the innate and adaptive immune systems in a phage specific manner (Dabrowska et al., 2014; Majewska et al., 2015) and potentially in a protein specific manner (Dabrowska et al., 2014). However, a number of studies performed with cocktails of whole phages have suggested that phages are harmless when ingested (Table 3; Bruttin and Brussow, 2005; Sarker et al., 2012) or applied topically (Rhoads et al., 2009). This lack of response may be in part due to the degradation of phages as they transit the digestive system, reducing the number that come into contact with immunostimulatory cells (Abedon, 2015), but also due to varying degrees of sensitivity between different cell types.

**Table 3:** **Current clinical trials for phage based therapy in humans**.

**Trial number**	**Study title**	**Status (date of completion)**	**Summary of trial**	**Published research articles**
NCT02664740	Standard treatment associated with phage therapy vs. placebo for diabetic foot ulcers infected by *S. aureus* (PhagoPied)	Not yet recruiting	• Multicenter trial comparing phage impregnated dressing (10^7^ PFU/mL) to a placebo dressing • Dressings to be replaced at Day 7 and 14 • Wants to recruit 60 participants • Measuring wound healing over 12 weeks • Presence/absence of bacteria and antibiotic resistance	–^c^
NCT02116010	Evaluation of phage therapy for the treatment of *Escherichia Coli* and *Pseudomonas aeruginosa* wound infections in burned patients (PHAGOBURN)	Recruiting (July 2016)	• Phase I/II multicenter trial comparing phage cocktails against Silver Sulfadiazine • Time taken to get a persistent reduction of bacteria relative to bacterial content at D0 • Assessing tolerance to the treatment • Assessing level of clinical improvement	–^a^
NCT01818206	Bacteriophage effects on *Pseudomonas aeruginosa* (MUCOPHAGES)	Completed (April 2012)	• Induced sputum samples taken from 59 CF patients • *Ps. aeruginosa* count after 6 and 24 h exposure to phage cocktail • Phage counts after 6 h	Saussereau et al., 2014
NCT00945087	Experimental phage therapy of bacterial infections	Unknown (Last Updated Sept 2013)	–^a^	
NCT00663091	A prospective, randomized, double-blind controlled study of WPP-201 for the safety and efficacy of treatment of venous leg ulcers	Completed (May 2008)	• Phase I safety study evaluating an 8 phage cocktail (each phage component approx. 10^9^ PFU/mL) • Desired enrollment of 64	Rhoads et al., 2009
NCT00937274	Antibacterial treatment against diarrhea in oral rehydration solution	Terminated (Jan 2013)	• Comparison of 2 separate T4 phage cocktails against standard oral rehydration solutions in ETEC and EPEC infections • Desired enrolment of 120 • Assessment includes safety tolerance and reduction of stool volume and frequency	Sarker et al., 2016

Data obtained from http://www.Clinicaltrials.gov (09/03/16).

a Not applicable.

Despite this initial suggestion of safety under Phase I conditions, potential safety issues will remain during Phases II–IV as “rarer” side effects are sought. At these stages, drugs under evaluation are subjected to trials of efficacy in a population who suffer from the particular disease under investigation. Due to the nature of the lytic phage lifecycle, it is not inconceivable that the active replication of phages at a site of infection could produce side effects, such as toxic shock, as bacterial debris is released. Such issues could potentially be anticipated and avoided by the selection of phages which exhibit different properties such as lower virulence or through the combination of phages with conventional antibiotics. Although this may suggest that whole replicating phage therapies could be consigned to topical application further research is required, particularly if the issue is addressed through the incorporation of anti-endotoxin adjuvants (de Tejada et al., 2015; Valera et al., 2015).

During Phase II and III studies additional complications arise when trying to recruit a statistically relevant homogenous population to study (Rose et al., 2014). In the case of diseases caused by a single bacterial species (e.g., cholera), this may be due to a low incidence in the general population or, and more likely, the disease can be caused by multiple organisms (e.g., diabetic foot ulcers). It is therefore likely that clinical trials on phages will be based on long term or multi-site studies in order to obtain representative population sizes and could be facilitated by the introduction of the EU-CTR. The introduction of the EU-CTR could also enable trials to be coordinated from specialist phage therapy centers allowing for the distribution of specialist knowledge and products.

Although the combination of multiple phages into a cocktail compensates for a limited host range (Bruttin and Brussow, 2005) a number of compromises are made. The increased complexity of multi-phage cocktails will dilute the concentration of the individual phage components due to their size, and also introduce competition of phages for binding sites, both of which could compromise the treatment (Nilsson, 2014).

Pre-made phage cocktails can be designed to target either against uncharacterized (multiple phages targeting multiple bacterial species), or typed bacterial infections (multiple phages targeting a single bacterial species). Additionally, patient specific cocktails can be produced in which phages are selected from a pre-existing library against the patient's specific strain (Pirnay et al., 2011). While the manufacture of pre-made cocktails would be tightly controlled and mass produced under cGMP in order to satisfy regulatory requirements (Parracho et al., 2012; Rose et al., 2014) it could decrease the production cost per dose. Pre-made cocktails would also require supplementary approval as cocktail components are modified to compensate for the development of bacterial resistance. The clinical usefulness of pre-made cocktails would be limited due to the shifting nature of epidemic strains; however, the rate at which resistance develops under therapeutic conditions is currently unknown. In theory at least, a pre-defined cocktail should be able to successfully navigate the current regulatory process assuming appropriate non-inferiority (drugs under investigation possess similar levels of activity compared to standard treatment) trial designs. However, it remains unclear if additional approval would be required as components of the cocktail change.

Although patient specific cocktails may provide better overall results (due to the tailored nature of the treatment) these would present additional challenges in order to gain regulatory approval. Classical trials of patient specific cocktails would have to be designed to target specified bacterial strains within the same species, further reducing the available population which could be recruited and require multi centered trials to be undertaken. In theory at least individual approvals would be required due to their unique composition. In order to compensate for the variety of potentially infectious strains, patient specific cocktails would require libraries of pre-approved phages to be developed. This would allow cocktails to be assembled on a case by case basis, currently an unprecedented move, although the stockpiling of vaccines and some antitoxins could be considered to be a suitable analog (Bodas et al., 2012; Martin et al., 2012).

As previously mentioned, the overall cost for the complete (pre-clinical to Phase III) development of a novel drug is astronomical (estimated to be US$2.6 billion; Mullard, 2014) and represents a significant obstacle for the broad evaluation of phage therapy in human populations. This cost would be on a per cocktail basis (assuming cocktails were pre-defined and manufactured) and probably equate to those encountered by an antibiotic or phage derived protein. When new strains arise, for which the pre-defined cocktail is not approved, treatment could still be carried out under compassionate usage or “off license.” However, in the case of patient specific cocktails, approvals may have to be obtained on a phage by phage basis, prior to combination into cocktails and could potentially, increase overall costs by orders of magnitude.

The biology and unique, but poorly understood, PD/PK of whole phage based therapies, may in actuality reduce their viability as antibiotic replacements for the treatment of bacterial infections in humans. In the case of PD, their large size and poor diffusion through non-aqueous mediums would present challenges if used as a systemic treatment. This would require potentially huge doses of phage to be administered in order to achieve a therapeutic effect. In terms of pharmacokinetics, large phage doses would be cleared efficiently from the body by the immune system and could prevent the establishment of a productive phage infection. The administration of large doses of phages would increase the probability of phages being able to reach the site of infection prior to being removed by the immune system. In addition to this, the overall immunostimulatory capacity of phage could be reduced through complex formulation by masking the phages (Kim et al., 2008) or through modification of phages to alter immunostimulatory proteins (Dabrowska et al., 2014).

Although many of the issues raised here have been presented as phase specific, they in fact transcend the individual trial phases and represent incompatibilities within the current approvals process itself. Indeed, for patient specific cocktails, it is highly improbable that current regulations would allow for the approval of a library rather than requiring the approval of each individual phage, drastically increasing the overall cost.

Conversely, phage derived products (e.g., endolysins) may address some of these limitations and have attracted some attention from commercial entities (Table 2). Despite being defined as “therapeutic biological products” their activity kinetics, and probably approval pathways, would be more akin to antibiotics than whole phage cocktails. The ability to produce them as recombinant proteins in a non-target vector means that overall manufacturing and purification processes could be adapted from currently existing methods (such as those used to produce insulin). This would allow for classification at a substantially higher level of detail than is possible with whole phage cocktails. The activity of endolysins has also been shown to transcend single bacterial strains in Gram positive pathogens such as *S. aureus* (Fischetti, 2016). This would not only enable trials to be performed on larger populations but could also increase their attractiveness to larger scale pharma companies, as they could be used to target multiple conditions caused by a particular bacterial species. However, they would be of limited effectiveness against polymicrobial infections. Many of the derived endolysins currently described in the literature target Gram positive pathogens (Fischetti, 2010; Nakonieczna et al., 2015). Further research is needed to evaluate their efficacy against Gram negative pathogens due to differences in cell wall composition as well as more research on Gram negative specific endolysins (Dong et al., 2015; Oliveira et al., 2016). Additionally, other phage derived proteins such as holins and tailspikes may provide suitable alternatives to endolysins for Gram negative pathogens (Saier and Reddy, 2015).

## Adaptive licensing frameworks

The process for gaining regulatory approval for novel antibiotics is a long and time consuming process, which is further complicated when whole phages are applied to these proceedings. Although the process itself is optimized for certain types of drugs, regulators believe that the current trials legislation is “adequate” for use with bacteriophage based therapies (Verbeken et al., 2012; Pelfrene et al., 2016). However, many researchers engaged in the field actively disagree with this, as the current approvals procedures are too rigid and too costly in terms of time and money. They have more recently suggested that current pathways need to be modified or novel pathways need to be developed for use with phages (Verbeken et al., 2014; Kutter et al., 2015; Young and Gill, 2015).

Multiple initiatives have been taken by both the FDA and Europe Medicines Agency (EMA) to simplify and shorten the approvals process for drugs while maintaining standards. They are not designed for antibacterial drugs or, more specifically, phage based therapeutics. These frameworks include an EMA pilot project on adaptive licensing initiated in 2014 (AL or adaptive pathways) and encompasses six undisclosed products. The pilot study, which is due to report later in 2016, seeks to investigate how current regulations can be optimized for the approval of new drugs in cases where there is a high medical need. The pilot also seeks to determine which criteria should apply for drugs that can be approved in a graduated simplified process.

Introduced to the US Senate in January 2015, the Promise for Antibiotics and Therapeutics for Health (PATH) Act (S.185; https://www.congress.gov/bill/114th-congress/senate-bill/185/text; accessed 13th June 2016) is an amendment to section 506 of the Federal Food, Drug, and Cosmetic Act (21 U.S.C. 356). This amendment proposes the creation of a “limited population pathway for antibacterial drugs” that will allow for the approval of antibacterial drugs within a highly defined population without the need for clinical trials through the development of a Benefit-Risk profile that reflects the “severity, rarity, or prevalence” of the infection. Although the decision making process could inevitably be informed by both traditional (e.g., survival), alternative (e.g., bacterial clearance), and small clinical data sets, the process may also take into consideration other supplementary information such as non-clinical susceptibility and pharmacokinetic data. However, the supplementary set of considerations will be decided on a drug by drug basis. Once approved, product labeling will reflect the limited population that the drug can be used on and subjected to post approval monitoring. Subsequent approval for use within a wider population can be sought, but it is not clear if this would require full clinical trials or if off license use would be permitted.

AL pathways are iterative processes in which treatment outcomes are used to inform the ongoing trial, through the involvement of all stakeholders, as well as input from independent scientific advice. The iterative process can, as in the EMA pilot project, be based on different conditions including: (i) the introduction of more steps, starting with a small highly defined patient population which is expanded as more information becomes available (ii) conditional approval for a product that is granted based on existing data, or (iii) adopting a centralized compassionate use of a new drug.

## Adaptive licensing for phages

When compared to classical trials, AL pathways may provide additional flexibility that would enable whole phage therapeutics and their derived products to be approved for clinical use. These opportunities would come in the form of initial limited population testing and potentially through the use of non-traditional surrogate endpoints.

**Figure 3:**
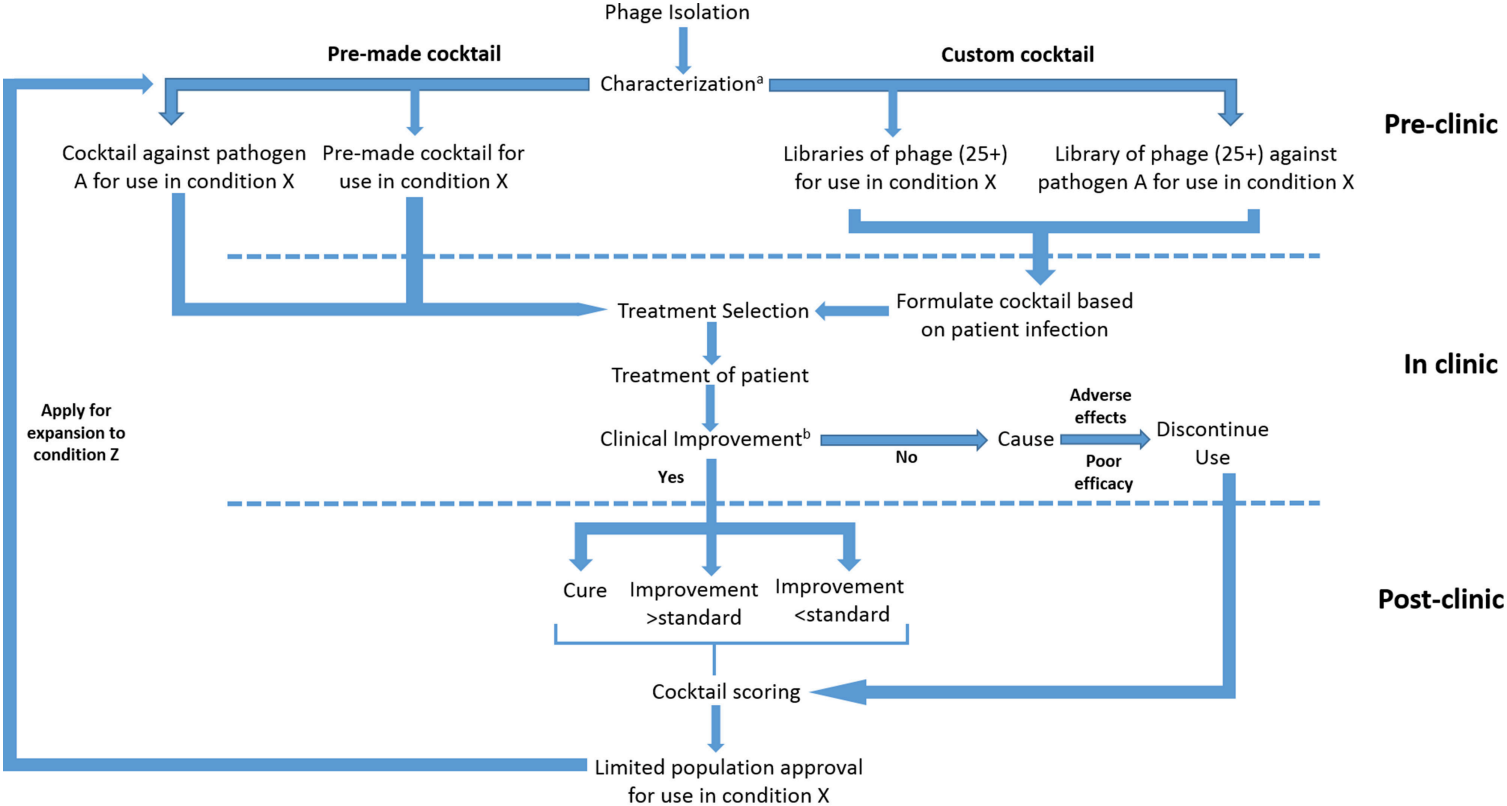
**Proposed submission of whole phage based products under an adaptive licensing framework**. ^a^Characterization to be based on genotypic analysis and lytic activity. ^b^Improvement to be characterized on the basis of classical and surrogate endpoints.

Regardless of the pathway employed, phage clinical trials will inevitably consist of a series of compromises due to the complex interplay between infection type, causative agent and therapeutic strategy. Trial outcomes will also shift depending on additional priorities such as clinical need, scope of the trial, anticipated efficacy of treatment, and overall cost. In addition to this, trials could be based around a multitude of different formulations, each possessing advantages and disadvantages (Figure 3).

## Form of therapy

As previously mentioned, phage therapy may involve treatment with single phages, phage derived products e.g., endolysins, or cocktails. Host range considerations would limit the available population that could be treated with single phage preparations, whereas the formation of phage cocktails would be able to increase the number of patients that could be treated. The number of potential recruits for trials could also be increased for disease states which cause seasonal outbreaks of a single clonal type in a confined area (e.g., hospital acquired *Clostridium difficilie* infections; Furuya-Kanamori et al., 2015). Trial participants could be easily recruited for clinical trials of a single phage, particularly if the target organism is a commonly occurring pathogen (e.g., MRSA). The recruitment process would be also be facilitated by a centralized approvals process (e.g., EU-CTR) which would enable multi-centered trials to be performed with a single application. However, many infecting bacterial strains are temporally or spatially restricted, limiting the availability of participants.

Phage cocktails, on the other hand, can increase the number of strains susceptible to infection by one or more of the components which make it possible to target common bacterial infections caused by different strains in different patients and thus facilitate the recruitment of participants. Cocktails can be pre-made and targeted against common pathogenic strains or custom made, either upon the emergence of a particular strain or to fit the requirements of individual patients (Pirnay et al., 2011).

### Pre-made cocktails

Highly defined pre-made cocktails should be able to fit into existing AL frameworks as it would be relatively simple to define a limited population, although this would not be able to compensate for differences between causative strains. Should the causative strain be specified (e.g., *Pseudomonas aeruginosa* PAO1) this would further limit the population which could be recruited, therefore a multi-centered approach would be needed and could be expedited by the modified EU-CTR. To compensate for shifting global trends, clinically relevant bacterial collections should be assembled and distributed which would enable easier isolation of suitable phages. It should also be noted that pre-defined cocktails are less flexible to the rise and fall of new bacterial strains and would ultimately be susceptible to the development of bacterial resistance. The overall efficacy would decrease over time in which a new cocktail would need to be developed and approved for use.

### Custom cocktails

Custom made cocktails are one way to address the development of bacterial resistance against phages. In the case of bacteria which harbor phage resistance systems (e.g., CRISPR), phages encoding specific anti-defense mechanisms e.g., anti-CRISPR systems (Bondy-Denomy et al., 2013) could be given higher priority even if their infection characteristics are not as good as other phages. Several phages targeting different surface receptors could be applied simultaneously or serially, resulting in a synergistic effect and could reduce the potential for resistance developing (Schmerer et al., 2014) although other criteria could be used for the selection of cocktail components (Chan and Abedon, 2012). The inducement of synergy between phages would also be a good strategy for long term treatment of deep infections. In such infections, phages would have difficulty reaching their targets and would be cleared by the immune system reducing their overall number. By inducing synergy a smaller overall number of phages would be required to reach the site of infection as each phage component would be able to establish a productive infection. However, as with all phage trials there are arguments that the overall number of participants in custom cocktail trials would be limited to just one (Eichler et al., 2015) as infecting strains, and therefore cocktail composition, would vary on a patient to patient basis. As such it may be advantageous to inform AL trials based on a pre-characterized library of phages against a defined pathogen in a defined condition (e.g., *Ps. aeruginosa* burn infections), although this would require a significant redesign of approvals processes.

### Creation of a pre-characterized library of phages and selection for use in patients

A long-term possibility for the implementation of whole phage therapeutics would be to create phage libraries containing the most efficient phages against the most severe pathogens, e.g., multiresistant Gram-negative bacteria. This could be done by initially pooling existing phage banks (most of them maintained by research institutions) into common libraries, followed by the continuous isolation and addition of new phages. Copies of the library would be stored in national phage depositories or major hospitals. This would shorten the time for finding highly efficient matching phages and assembling cocktails as well as facilitate an approval process. Individual phages would initially be characterized *in vitro* (as with most isolation and characterization papers) using structural, genomic (i.e., absence of lysogenic properties), host range, and efficacy analyses (Malki et al., 2015; Sauder et al., 2016). However, and more importantly, the pre-characterization could also establish safety and efficacy including suitable *in vivo* testing in animal models before phages are added to the library. It is important to ensure that even phages which do not meet the required *in vitro* efficacy criteria (e.g., insufficient lytic activity) are not discarded as they may perform better *in vivo* (e.g., less immunostimulatory), or possess greater efficacy against future epidemic strains.

The assembly of pre-approved phage libraries could potentially prove advantageous as it would allow for the creation of multiple cocktails to target an individual infection type within a single library thereby increasing the population size that could be recruited. Only the cocktails themselves would need to be tested for safety and efficacy since individual phages in the library would already be fully tested. This approach would also allow for the creation of additional libraries for phages which appear to be less active or possess a restricted host range. Indeed the possibility of creating multiple or tiered libraries (as is the case with multiple lines of antibiotics) would allow additional flexibility as resistance to individual phages develops. However, as the complexity of cocktails increases to treat polymicrobial infections, or as multiple tiered libraries are assembled, the overall cost and time required to complete trials would increase.

It should be noted that the concept of pre-approved libraries would require a radical shift in the thought processes of regulatory agencies and would require the development of new assessment criteria. These criteria could include the absence of non-toxicity regardless of the combination of phages used, in combination with a “minimal” activity level of each phage. Such a shift in thought process would also ultimately lead to the development and approval of libraries of phage derived antibacterial proteins. By approving both phages and their derived proteins as libraries rather than on an individual basis, the overall number of trials would be reduced due to increased flexibility of the drug and potentially an increased treatment success rate.

### Patient criteria and emergency procedures

Following the successful formation of a pre-characterized library or specified cocktail, patients would be recruited on the basis of confirmed infection type (i.e., a specified bacterial agent in a specified disease state). As in the EMA pilot studies, the trial would progress iteratively, starting with small groups of participants and resolving uncertainties before expanding the trial into new populations. Adverse effects influenced by rare human genetic traits may be problematic for this type of AL approach, but further research is required. As a consequence, the initial (human) testing of phages or cocktails from the libraries should be conducted in non-life threatening topical infections (e.g., diabetic foot infections). Any target infection should also have at least one additional treatment available as a safety precaution. Should the application of the cocktail result in Serious Adverse Events/Serious Adverse reactions, Suspected Unexpected Serious Adverse Reactions or no measurable clinical benefit, the decision to remove a patient from the treatment should be made quickly and alternative treatment applied as soon as possible.

### Assessing outcomes and expansion of populations

The necessary expansion from small groups of trial participants to larger cohorts during adaptive pathway trials would be contingent on the outcomes that can be assessed given the actual cohort size. Classical clinical trials often use well-defined and clinically relevant endpoints like patient survival time, resolution of infection, decrease in lesion size or perceived symptoms, but surrogate endpoints based on biomarkers for indirect assessment can also be applied (Fleming and Powers, 2012).

In the initial stages, classical endpoints should be included in adaptive pathway phage therapy trials, but pharmacological assessment will be as important. Surrogate endpoints will probably be of greater importance for phage therapy trials. Increase in phage titers, reduction of bacteria load or infection parameters (e.g., CRP) and absence of additional pro-inflammatory responses may indicate that the treatment has a positive effect and that the study can be widened. Therefore, phage trials under AL would be designed as non-inferiority trials, in which the intervention is compared to the conventional therapy (e.g., antibiotics) to establish a similar level of overall effect (D'Agostino et al., 2003) rather than having to demonstrate superiority.

If applied in a pre-formed library format for patient specific cocktails, individual phage components could potentially be scored to further inform and develop the library. Such a scoring mechanism would not only require the treatment outcome to be established, but the actual role of the individual phage in that outcome and would require the ability to differentiate between the components of the cocktail.

AL pathways offer many possibilities for the approval of whole phages. However, each of these different avenues require compromises which will subsequently impact the efficacy of the treatment and on the overall cost and time required to complete trials (Table 4). In the case of cocktails the time and cost to reach the clinic will increase significantly if it is necessary to approve each individual component of the cocktail separately. However, if radical action is taken and libraries of phages against specified pathogens are approved, this could potentially counter the increased cost and time.

**Table 4:** **The implementation of adaptive licensing pathways for single phage and pre-made or custom phage cocktails**.

	**Single phage**	**Pre-made cocktails**		**Custom cocktails**		
		**Untyped infections**	**Typed infections**	**Single species typed infections**	**Multi-strain typed infections**	**Polymicrobial typed infections**
Phage selection	• One phage with high efficacy against a typed bacterial strain.	• Multiple phages targeting an untyped infection.	• Multiple phages, compromising between efficacy and host range.	• Multiple phages targeting a single typed bacterial strain.	• Multiple phages targeting multiple typed bacterial strains.	• Multiple phages targeting multiple typed bacterial species.
Aim	• Treat MDR/XDR strains only.	• Treat bacterial infections based on symptoms.	• Treat infections caused by multiple strains of the same species.	• Treat patient specific infections caused by a single typed species.	• Treat multiple strains of the same species in a single patient.	• Treat typed polymicrobial infections in a single patient.
Advantages	• Easy to change phages. • Easy analysis.	• Easy recruitment to trials.	• Easy recruitment to trials. • Only limited phage library needed.	• Predicted high efficacy. • Avoids emerging bacterial resistance.	• Wide application.	• Wide application.
Disadvantages	• New phages require additional trials. • Unpredictable results.	• Poor patient recovery rate. • Unpredictable results. • New formulation means new trial.	• Cocktail obsolescence. • New formulation means new trial.	• Unique cocktails increase recruitment difficulty to trials. • Cocktail obsolescence. • Large phage library needed.	• Every cocktail unique, more difficult to recruit trial participants. • Necessary to reformulate cocktail as new strains appear. • Large phage library needed.	• Every cocktail unique, more difficult to recruit trial participants. • Necessary to reformulate cocktail as new strains appear. • Multiple large phage libraries needed.
Iteration	• Phage is discarded or formulation changed as new data emerges.	• Non-effective or hazardous phages are removed as data emerges. • Additional phase I testing if there is poor efficacy or immunogenicity.	• Non-effective or hazardous phages are removed as data emerges. • Additional phase I testing if there is poor efficacy or immunogenicity.	• Cocktail composition altered as data on phages emerge. • Additional phase I testing if there is poor efficacy or immunogenicity.	• Cocktail composition altered as data on phages emerge. • Additional phase I testing if there is poor efficacy or immunogenicity.	• Cocktail composition altered as data on phages emerge. • Additional phase I testing if there is poor efficacy or immunogenicity.
Design	Single site long term or multi-site short term	Single site long term or multi-site short term	Single site long term or multi-site short term	Multi-site long term	Multi-site long term	Multi-site long term
Implementation time	+	+	+	++ or +++^*^	++ or +++	+++
Cost	$	$$	$$	$$$	$$$	$$$

* Time to implementation would be affected by the form of treatment chosen. Pre-approved libraries could be implemented faster should suitable criteria be developed.

Although it is likely that phage derived proteins will not suffer adversely when trying to gain regulatory approval, AL pathways could still prove beneficial. The use of a limited population approach would enable data to be obtained while informing future clinical studies of different disease states, particularly if developers are interested in systemic application. In addition to this, classical and surrogate endpoints could be utilized that could be derived from antibiotic trials (Cornely et al., 2012; Verduri et al., 2015).

## “Right to try” legislation and “compassionate use”

The Code of Federal Regulations (CFR) Title 21, Chapter I, Subchapter D, Part 312, Subpart I (http://www.ecfr.gov/cgi-bin/text-idx?SID=43f054659224216924a6379ef9602c2b&mc=true&tpl=/ecfrbrowse/Title21/21tab_02.tpl; accessed 13th June 2016**)** and European Regulation 726/2004/EC (http://eur-lex.europa.eu/LexUriServ/LexUriServ.do?uri=OJ:L:2004:136:0001:0033:en:PDF; accessed 13th June 2016) govern the expanded access of non-approved medications to patients that have been submitted for approval by regulatory agencies. Although this is often under compassionate use guidelines where no alternative treatment exists (Whitfield et al., 2010) there are increasing calls to amend the laws in the US to make this process easier through additional legislation such as the H.R 3012 (Right to Try Act; https://www.congress.gov/bill/114th-congress/house-bill/3012; accessed 13th June 2016). Introduced to Congress during July 2015, H.R. 3012 would allow Phase I experimental drugs, biological products, or devices to be used in terminally ill patients.

Although such regulations would allow for the use of phages on individual patients in the US under Part 312.310, expansion to an intermediate patient population size (Part 312.315) would be possible, but potentially difficult. While there have been a limited number of cases in which phage cocktails have been approved for compassionate use this process is not routine (Rhoads et al., 2009; Khawaldeh et al., 2011). However, should resistance rates continue to increase, and the number of available drugs decrease further, phage therapy may be the only remaining therapeutic option.

In addition to cGMP and other specific requirements, informed consent from patients is required for compassionate use. While this should be relatively simple, the lack of public awareness surrounding phages may be detrimental to recruitment. This lack of public awareness could be circumvented by the formation of trials in EU member states possessing specialized phage centers (such as the Institute of Immunology and Experimental therapy in Wroclaw Poland) as part of an EU-CTR application in combination with compassionate usage in other parts of Europe which has been suggested by researchers in the field (Kutter et al., 2015).

## Utilization of additional data sources

The clinical use of phages in specialized centers in Eastern Europe has generated immense amounts of data, little of which is published in Western scientific literature (Miedzybrodzki et al., 2012). As some of these centers now lie within the EU, data generated post membership should conform to Western standards and could potentially form the basis of a meta-analysis or systematic review of clinical phage therapy when combined with more recent trial data that has been generated.

In order to utilize such data that has been generated prior to EU membership, or for countries whose regulatory frameworks may differ to EU and FDA standards, criteria would need to be established in order to assess the overall quality of the work performed. This could be done on the basis of the achievement of an appropriate clinical outcome (i.e., duration of hospitalization or resolution of infection) or an assessment of the methodology and trial design prior to incorporation into meta analyses.

## Concluding remarks

Despite the pressing need to develop new antibacterial agents, the approval rate of anti-bacterial drugs remains low when compared to other forms of drug due in part to both economic and scientific issues. Phage derived products, such as endolysins, are likely to be suitable for classical clinical trials procedures due to their similarities with conventional antibiotics. However, in the case of whole phage therapies, currently available mechanisms are not suitable, requiring large patient cohorts and extensive resources. In this article the limitations of current clinical approval pathways, as well as possible alternative pathways for the approval of phage therapy, have been discussed and summarized (Table 5).

**Table 5:** **Summary of current and possible alternative pathways as applied to whole phage and phage derived therapeutics**.

	**“Classical” licensing**	**Adaptive licensing**	**Compassionate use**
Advantages	• “Gold” Standard • Already established for antibacterial drugs • Would be suitable for phage derived products • Additional legislation being introduced to streamline procedures for antibacterial drugs	• Limited population approvals • Iterative process which can inform future work • Can be adapted for pre and custom phage cocktails	• Immediate clinical usage • Data could be used to inform future work • Could be utilized for all forms of phage therapy
Disadvantages	• Recruitment for trials • Cost • Reformulation would require additional trials	• Varying degrees of complexity • Limited population approvals	• Limited to a single patient basis • Not actually approved for use
Other considerations	• Likely that only highly defined products would be able to succeed, limiting success	• Approval of predefined libraries would require wholly new approvals process	• Lack of public awareness of phages
Time to implement^a^	++	++ or +++^*^	+
Cost to implement^a^	$$$	$$ or $$$^*^	$

a Assumption has been made that “Classical” licensing is a baseline.

* Both cost and time to implementation would be affected by the form of treatment chosen. Pre-approved libraries taking longer and costing more to achieve.

While the current article is by no means exhaustive on every potential pathway that could be employed for the approval of phage based therapies for human use, it hopefully sparks discussion and debate on the nature of clinical trials and the need for more flexible regulations when dealing with phages and their derived products. For phages that are genetically similar (>95%) this could include additional accelerated or automatic approval pathways. This would be particularly useful for those phages whose major components (e.g., capsids) are identical but whose host range and efficacy is influenced by small changes in tail fiber composition (Ando et al., 2015; Goren et al., 2015). However, genetic engineering of whole phages (Ando et al., 2015), or the creation of wholly artificial phages from sequence data (Smith et al., 2003), would require approvals pathways to be revisited in the future as these technologies approach clinical readiness. In the case of customizable cocktails taken from pre-licensed libraries, suitable regulatory criteria need to be developed. This would in essence separate phage approvals from the normal biological therapeutics approvals and would require a substantial shift in the collective mindset of regulatory agencies.

Perhaps the most radical possibility would be to establish a centralized phage bank under governmental control from which phages could be isolated, collated, tested, and distributed on a case by case basis to be used in compassionate use treatments or AL trials. Data and treatment outcomes could then be collated by the phage bank to provide greater insight into phage therapy as a whole. This would not only remove the commercial element to development, but would provide direct to clinic access for therapies and also enable a greater degree of control to be exerted over treatment potentially reducing phage resistance rates. Such a system could be developed within existing public health organizations (e.g., Public Health England, UK or Folkhälsomyndigheten, Sweden) as these organizations are responsible for collating data on antibiotic resistance trends and provide reference laboratory facilities, but would require a substantial initial investment to establish and thus may be unpalatable in the current economic climate.

While no phage specific approvals pathway currently exists, such a pathway could be developed with suitable engagement between regulators and researchers. This pathway could be based on existing guidelines, where products which have been successfully completed phase I clinical studies are applied on a case by case basis under compassionate use guidelines. However, in order to gain widespread adoption it may be better to base phage approvals on AL principles, whereby approval is granted for small specified populations which can then be expanded upon as post-approval data is gathered.

Regardless of the pathway implemented, the overall cost of drug development and the poor return on investment of antibacterial agents will remain one of the defining development issues. Due to the abundance of phages in the environment, patents may be circumvented relatively easily as new phages are isolated and would therefore reduce the potential level of interest from traditional pharmaceutical companies.

## Author contributions

All authors listed, have made substantial, direct and intellectual contribution to the work, and approved it for publication.

### Conflict of interest statement

The authors declare that the research was conducted in the absence of any commercial or financial relationships that could be construed as a potential conflict of interest.

## Footnotes

^1^ASTM E2197-11, Standard Quantitative Disk Carrier Test Method for Determining the Bactericidal, Virucidal, Fungicidal, Mycobactericidal and Sporicidal Activities of Liquid Chemical Germicides, ASTM International, West Conshohocken, PA, (2011), www.astm.org.
